# It’s time to change our documentation philosophy: writing better neurology notes without the burnout

**DOI:** 10.3389/fdgth.2022.1063141

**Published:** 2022-11-28

**Authors:** Jorge M. Rodríguez-Fernández, Jeffrey A. Loeb, Daniel B. Hier

**Affiliations:** ^1^ Department of Neurology, University of Texas Medical Branch, Galveston, TX, United States; ^2^ Department of Neurology and Rehabilitation, University of Illinois at Chicago, Chicago, IL, United States; ^3^ Department of Electrical and Computer Engineering, Missouri University of Science and Technology, Rolla, MO, United States

**Keywords:** electronic health records, documentation burden, clinician well-being, evaluation and management coding, medical decision-making

## Abstract

Succinct clinical documentation is vital to effective twenty-first-century healthcare. Recent changes in outpatient and inpatient evaluation and management (E/M) guidelines have allowed neurology practices to make changes that reduce the documentation burden and enhance clinical note usability. Despite favorable changes in E/M guidelines, some neurology practices have not moved quickly to change their documentation philosophy. We argue in favor of changes in the design, structure, and implementation of clinical notes that make them shorter yet still information-rich. A move from physician-centric to team documentation can reduce work for physicians. Changing the documentation philosophy from “bigger is better” to “short but sweet” can reduce the documentation burden, streamline the writing and reading of clinical notes, and enhance their utility for medical decision-making, patient education, medical education, and clinical research. We believe that these changes can favorably affect physician well-being without adversely affecting reimbursement.

## Introduction

A crisis in physician well-being and the mounting burden of clinical documentation drives the need for neurologists to change their documentation philosophy. Burnout is prevalent among all healthcare professionals (1) and neurologists in particular (2, 3). Documentation is a known contributor to burnout (1, 4, 5). An estimated 40% of physician time in the electronic health record (EHR) is devoted to documentation (6). In 2015, the American College of Physicians (7) emphasized the importance of writing “concise, history-rich notes” that prioritize information relevant to medical decision-making (MDM). Responding to physician complaints about the burden of EHR documentation, the AMA Current Procedural Terminology Editorial Panel recommended changes to evaluation and management (E/M) documentation guidelines that would reduce the clerical burden (8). On January 1, 2021, the Center for Medicare and Medicaid Services (CMS) updated E/M coding guidelines for outpatient visits that would allow physicians to select a billing level based on the time or complexity of MDM and would reduce documentation requirements for the history and physical examination (including the unpopular bullet point requirements). These requirements were seen as both tedious and time-consuming. Furthermore, they were seen as reducing the time available for direct patient care (6). CMS plans to extend these changes to the inpatient setting (hospital inpatient, hospital observation, emergency department, and cognitive impairment assessment) by January 1, 2023 (9).

Time-based coding allows physicians to bill for time spent outside the patient visit on the day of evaluation, even if the patient is absent (chart review, documentation, orders, coordinating care, etc.) The complexity of medical decision-making (the main driver of billing level) is determined by the number and complexity of the problems addressed, the risk of complications (morbidity and mortality), and the complexity of the data reviewed. These changes simplify setting the correct level of service for billing purposes. For example, a patient with migraine headaches who is started on single-drug therapy has a low level of MDM; a patient with new-onset seizures who needs adjustments of anti-epileptic medications and imaging has a moderate level of MDM; and a patient with epilepsy and poorly-controlled seizures, a structural brain lesion, and who needs neuroimaging, extended electroencephalographic testing, and a surgical consultation has a high-level of MDM.

Initial studies suggest that these CMS changes will enhance reimbursement for Evaluation and Management (E/M) services (10) provided by non-surgical specialties but not by surgical specialties (11). Improvements have not yet been noted in time spent documenting in the EHR (10). Furthermore, to date, no study has determined that these changes in documentation guidelines have lightened provider documentation burden.

## Why are our notes so bulky?

In the US, the volume of clinical documentation has increased over the past two decades. It is estimated that US clinicians do three times more documentation than clinicians in other medically advanced countries (12). The are several reasons for the bulkiness of our clinical notes in the US.

• We have been reimbursed more for bulkier notes. Since 1995, the level of service and reimbursement was linked to the number of coding elements (also known as “bullet points”) documented (9).
• It’s too easy to add bulk. Copy and paste functionality in electronic health records makes it easy to add bulk. Hyperlinks make it possible to add laboratory results, radiology findings, problem lists, medication lists, and other chart elements to a note with a single mouse click. One study of over 26,000 physician notes found that only 18% of the text was entered by the physician, 46% was copied from elsewhere in the EHR, and 35% was imported from other sections of the EHR (13). Copy and paste may document care that was never rendered or examinations that were never performed (14).
• We are trained to document negative findings. As Sinsky has observed, we need to move away from the dictum that “If it wasn’t documented, it wasn’t done” (15). Or, as Postal has wondered, do we have the time and energy to document all the negatives or should we stick to the salient positives (16)?
• We continue to document based on reimbursement and tradition rather than scientific evidence. In their essay subtitled “A farewell to the review of systems,” Barry and Tseng argue for deleting the traditional review of systems and that documentation should be based on scientific evidence (17).

## It is time to change our documentation philosophy

A change in documentation philosophy is needed to slim down bloated notes that are hard to read, hard to write, and often inaccurate (Table 1). Copying and pasting text from one note into another fosters bloat, redundancies, and inaccuracies. When long pre-completed templates are used to document the neurological exam, parts of the neurological examination may be documented as normal when these parts were not examined. These documentation practices open the door to litigation. When test results (imaging, electroencephalography, electromyographic, etc.) are added to clinical notes, they should be addressed, discussed, and made relevant to the MDM.

**Table 1:** Suggested changes in documentation philosophy.

Old philosophy	New philosophy
Bigger is better	Less is more
	Short but sweet${}^{a}$
If it is not documented, it did not happen	Avoid excessive documentation of normal findings
Document all pertinent negative findings	Focus on abnormal findings
Import labs, radiology, allergies, medications, family history, and social history into every note	Maintain histories in one up to date central location
Longer notes with more bullet points are reimbursed at higher levels	Reimbursement is focused on medical decision-making
Documentation is the responsibility of the physician	Documentation is a team responsibility
Each physician is free to document as they please	Let’s agree on a uniform approach to documentation
Patients will not read our notes	Patients can benefit from reading our notes
Notes are for patient care	Notes can be used for research and patient care

${}^{a}$Especially in neurology where the history of the event and quantitation of symptoms is often more important to make the diagnosis than MRI imaging and other testing. A detailed, chronological description of symptoms is critical to diagnostic accuracy in neurology.

Documentation needs to be clear, accurate, and concise. The emphasis should be on optimal patient care, not maximizing the billable level of care. Neurology departments need to discourage clinicians from the redundant documentation of information. We argue that certain types of medical data (e.g., social history, past medical history, allergies, surgical history, medication lists, laboratory results, and radiology results) are best housed in a central location in the EHR and should not be added to a note unless relevant to MDM. Clinicians need to be trained to document allergies, medications, past medical history, and social history in the appropriate place in the EHR and not redundantly in each note. This practice has multiple advantages: it allows patient care team members to share the work of documentation, it reduces duplicate work, and it reduces multiple and inconsistent versions of the same data.

## We can build consistency and accuracy of documentation through standardized notes with quantitative longitudinal measures

Notes that have a consistent structure across the organization make notes easier to read and more predictable. Having a single consolidated note template for each department or subspeciality facilitates note maintenance. Detailed documentation of the history or the condition needed by a subspecialist can be collapsed in the EHR or linked elsewhere to not overwhelm the generalist user. In addition to text, developing innovative ways to visualize changes quantitatively in symptoms over time and their response to treatments can enhance the clinician’s perspective on the disease course. An additional benefit of a single template is the option of providing organizational updates or reminders to all template users.

The general SOAP (subjective-objective-assessment-plan) format has been well accepted. With the growing emphasis on MDM, the APSO (assessment-and-subjective-objective) format has grown in popularity. We additionally recommend the following:

• Create an institutional culture that values concise information-rich notes.
• Encourage clinicians to use collapsible sections in their notes to prioritize which sections are visible.
• Standardize the adoption of the SOAP or APSO note format at the organizational level.
• Encourage providers to document pertinent negative and positive findings through direct entry into the EHR rather than by template or copy and paste.
• Discourage providers from using hyperlinks in the EHR to add laboratory and ancillary testing results to notes with unnecessary redundancy when not relevant to MDM.
• Encourage providers to focus on adding those findings to notes that are pertinent to medical decision-making.
• Implement “vanishing text” that allows clinicians to view findings in their notes to support note creation and have it deleted when the note is finalized.
• Discourage the use of pre-completed examination templates with all findings marked as “normal.”
• If radiology or other reports are incorporated into a note, encourage the insertion of the “Impression” paragraph only.
• Look for help from the facility informatics department to create space-saving ways to represent bulky laboratory results as “fish bones” and other laboratory diagrams.
• Use hyperlinks, rather than text insertion, to connect notes to discrete data such as advanced directives or resuscitation status.
• Develop and implement policies that control copy and paste functionality, including highlighting of text that has been pasted into the note (14, 18).
• Use EHR metrics such as *note length*, *time in chart*, etc., to track changes in documentation practices by clinicians.

## Let’s engage providers and leadership in positive changes

The implementation of these recommendations depends upon proper organizational support. The engagement of key stakeholders, including departmental leadership, compliance officers, billing, coders, clinicians, and clinical informaticians, is critical. Principles and objectives for documentation change must be developed, agreed upon, and implemented. Documentation metrics are crucial to evaluating project success. Key documentation metrics include clinician time in the EHR, clinician time spent documenting outside of regular work hours, and time spent writing notes (19). We additionally suggest tracking mean note length over time. These metrics can demonstrate project success tangibly to leadership and clinicians.

The use of sprints or PDSA (plan-do-study-act) cycles can address implementation barriers before and after project roll-out. Departmental support is critical to assisting physicians in adjusting to new documentation methods. Iterative sprints and cycles are recommended to foster change. Although some documentation changes are driven by CMS, other regulatory agencies might have specific documentation requirements that require compliance, such as quality measures related to stroke (20). Interdisciplinary teams tasked with documentation change must address documentation compliance issues for each sub-specialty.

## Let’s get ready for OpenNotes

OpenNotes is coming. Federal legislation under the 21st Century Cures Act provides patients access to notes without delay and charge by April 5, 2021 (21). The OpenNotes initiative seeks to provide patients with access to their medical records (22) to improve their understanding of their condition and give patients more control of their treatment plan. Open communication with the patient during the visit combined with succinct understandable notes supports co-ownership of medical problems by patient and clinician (23–28). Long-term outcomes from the OpenNotes initiative on patient satisfaction and patient condition are still being evaluated.

As neurologists, we must recognize that patients will be reading our notes. For some sensitive conditions, this may be problematic. For example, in neurology, we often evaluate patients with functional disorders who have a limited understanding of the causes and nature of their condition. Neurologists must be open and should provide transparent communication with the patient at the time of evaluation and while creating their notes. Patients with functional disorders may find terms such as “non-physiological” or “no neurological correlate” confusing. These patients deserve a clear explanation of their symptoms in the office and in our notes (29, 30) Lastly, concerns on disclosure and result interpretation of sensitive testing for diseases such as Alzheimer’s disease have been raised, and efforts to develop ethical and patient-centered policies for disclosure are needed (31).

## Let’s embrace team-based documentation

The goal of team-based documentation is to offload some of the documentation work from the physician to other team members. Team-based documentation may variably involve scribes, nurses, pharmacists, medical assistants, or artificial intelligence-based dictation devices (32). Some team-based care models distribute specific documentation tasks such as recording allergies, documenting past medical history, and reconciling medication to specific team members (33). When these tasks are done before the initial interaction between patient and clinician, clinician time and effort are conserved. Other team-based documentation models use scribes to free up clinician time at the point of patient contact (32).

## We can flex our documentation to support education

Clinical documentation is central to the education of medical students, residents, and fellows (34). Although uniformity in documentation templates and methods is a stated goal, it is important to flex documentation expectations according to the level of training. Although it is reasonable to expect a medical student to document their neurological examination and history in great detail, the same is not true for an advanced fellow or experienced attending neurologist. While longer notes are de rigeur for medical students, we expect experienced clinicians to write concise notes with few notations about normal findings. Still, we should encourage medical students to focus on succinct formulations of the neurological examination and history. Academic institutions can take advantage of current E/M guideline changes to encourage trainees to document concisely, to prioritize MDM, and to avoid adding uninformative “bullet points” (35, 36). Documentation metrics can guide trainees and their mentors to adopt the best documentation strategies.

## Clinical notes can support research

Although the primary purpose of physician notes in the EHR is to document care rendered, to support the billing for services provided, and to serve as a medical-legal record; electronic health records and free text physician notes have shown great potential for clinical research (37–39). For example, EHRs are being used to track disease severity, and progression in cohorts of patients with amyotrophic lateral sclerosis and multiple sclerosis (40–42). Natural language processing and other artificial intelligence algorithms are unlocking latent value in EHRs. Unlike skimpy information-poor notes or bloated information-poor notes, concise information-rich clinical notes can be of great value for clinical research.

## Conclusion

For over two decades, our clinical notes have grown too long. They are a significant burden and contribute to physician burnout. This Perspective describes recommendations to simplify documentation that can be implemented because of changes in CMS guidelines for evaluation and management coding and billing. We argue that it is time to rethink our documentation to enhance communication, improve patient care, and reduce physician burnout. Although more work is needed to find optimal strategies to reduce the documentation burden, it is not too early to start creating notes that are easy to write and read yet are still information-rich. Bulky notes waste the time of the writer and the reader alike. Evidence is growing that when concerted efforts are made to simplify documentation, physician satisfaction with the EHR improves, and burnout is reduced (43–45).

## Data Availability

The original contributions presented in the study are included in the article/Supplementary Material, further inquiries can be directed to the corresponding author/s.
